# Climate sensitivity of seasonal radial growth in young stands of Mexican conifers

**DOI:** 10.1007/s00484-022-02312-3

**Published:** 2022-06-08

**Authors:** Marin Pompa-García, J. Julio Camarero, Cristina Valeriano, Eduardo D. Vivar-Vivar

**Affiliations:** 1 Facultad de Ciencias Forestales y Ambientales de la Universidad Juárez del Estado de Durango, Rio Papaloapan Y Blvd. Durango S/N. Col. Valle del Sur, 34120 Durango, Mexico; 2 Instituto Pirenaico de Ecología (IPE-CSIC), Avda. Montañana 1005, 50192 Zaragoza, Spain

**Keywords:** Dendroclimatology, Drought stress, Earlywood, *Pinus pinceana*, VS-Lite model

## Abstract

Alteration of forest by climate change and human activities modify the growth response of trees to temperature and moisture. Growth trends of young forests with even-aged stands recruited recently when the climate became warmer and drier are not well known. We analyze the radial growth response of young conifer trees (37–63 years old) to climatic parameters and drought stress employing Pearson correlations and the Vaganov-Shashkin Lite (VS-Lite) model. This study uses tree rings of six species of conifer trees (*Pinus teocote*, *Pinus pseudostrobus*, *Pinus pinceana*, *Pinus montezumae*, *Pinus ayacahuite*, and *Taxodium mucronatum*) collected from young forests with diverse growth conditions in northern and central Mexico. Seasonal ring growth and earlywood width (EW) were modeled as a function of temperature and soil moisture using the VS-Lite model. Wet and cool conditions in the previous winter and current spring enhance ring growth and EW production, mainly in sensitive species from dry sites (*P. teocote*, *P. pseudostrobus*, *P. pinceana*, and *P. montezumae*), whereas the growth of species from mesic sites (*P. ayacahuite* and *T. mucronatum*) shows little responsiveness to soil moisture. In *P. ayacahuite* and *T. mucronatum*, latewood growth is enhanced by warm summer conditions. The VS-Lite model shows that low soil moisture during April and May constrains growth in the four sensitive species, particularly in *P. pinceana*, the species dominant in the most xeric sites. Assessing seasonal ring growth and combining its response to climate with process-based growth models could complement xylogenesis data. Such framework should be widely applied, given the predicted warming and its impact on young forests.

**Supplementary Information:**

The online version contains supplementary material available at 10.1007/s00484-022-02312-3.

## Introduction

The world forests are experiencing the recurrent impacts of droughts that affect their productivity, composition, and structure (Maes et al. 2019). Multiple evidences of forest dieback and growth decline have been reported associated to dry spells (Allen et al. 2015). Therefore, the future facing forests is still an open question for the scientific community (Marchand et al. 2019). Solving this question is essential for forest productivity forecasting, especially in drought prone areas such as north and central Mexico where climate models forecast warmer and drier conditions (Seager et al. 2007).

In north and central Mexico regions, many conifer species coexist, thus offering a unique opportunity to improve the understanding of ecological changes in different forest types (Halffter and Morrone 2017). These diverse forests provide multiple ecosystem and economic goods and services to local populations (Farjon and Styles 1997). Previous studies showed the climatic responsiveness of tree growth in some of these Mexican conifers with a focus on radial growth sensitivity to drought (e.g., Villanueva-Díaz et al. 2007; Stahle et al. 2016; Pacheco et al. 2020; Correa-Díaz et al. 2020). However, these studies must be refined using seasonal ring growth data to obtain a more realistic understanding on the climatic constraints of tree radial growth.

One strategy to study seasonal ring growth in conifers is to subdivide intra-annual changes in radial growth into earlywood (EW) and latewood (LW) production (e.g., Meko and Baisan 2001). The earlywood formation is related to winter-to-spring climate variability, and it reflects the period with highest growth rates (Vaganov et al. 2006). Latewood production is driven by summer-autumn moisture in seasonally dry areas (Howard et al. 2021).

To properly assess how earlywood is limited by water shortage, both atmospheric water demand and soil moisture dryness should be also considered. These variables related to drought stress may be linked to in process-based growth models such as the Vaganov-Shashkin Lite (VS-Lite) model, which provide a better understanding on the climatic limitations of tree growth (see, for instance, Tolwinski-Ward et al. 2011; Sánchez-Salguero et al. 2017a, b). To the best of our knowledge, this model has been applied to tree-ring width data but not to earlywood production.

Furthermore, there are few studies modeling growth responses to climate focusing on young stands (but see Fonti et al. 2018), which are representative of current tree populations recruited under global-change conditions, i.e., elevated atmospheric CO_2_ concentrations and warm, and often dry, conditions. Mature or old trees growing in harsh sites with low soil moisture availability have been sampled to enhance the climate signal in studies carried out in northern Mexico (see Pompa-Garcia et al. 2021). In boreal forests, *Picea glauca* trees became increasingly moisture stressed with age confirming that age-dependent responses to climate may be found in different biomes (Szeicz and MacDonald 2011). Thus, determining the climatic parameters limiting seasonal radial growth in young stands should be a research priority to forest ecologists (e.g., Alfaro-Sánchez et al. 2019).

This study assesses the growth response to climate of six conifers species widely distributed across geographical and climatic gradients in north and central Mexico. This assessment is based on (i) correlations calculated between seasonal ring growth and climate and (ii) simulations based on the VS-Lite model and focusing on limitations of growth by low soil moisture. We hypothesize that (i) earlywood production will reflect the responses of young forests growth to low soil moisture conditions and (ii) there would be differential growth responses among species with higher growth sensitivity in species from xeric sites with variable year-to-year precipitation.

## Methods

### Study area, tree species, and sampling strategy

**Fig. 1:**
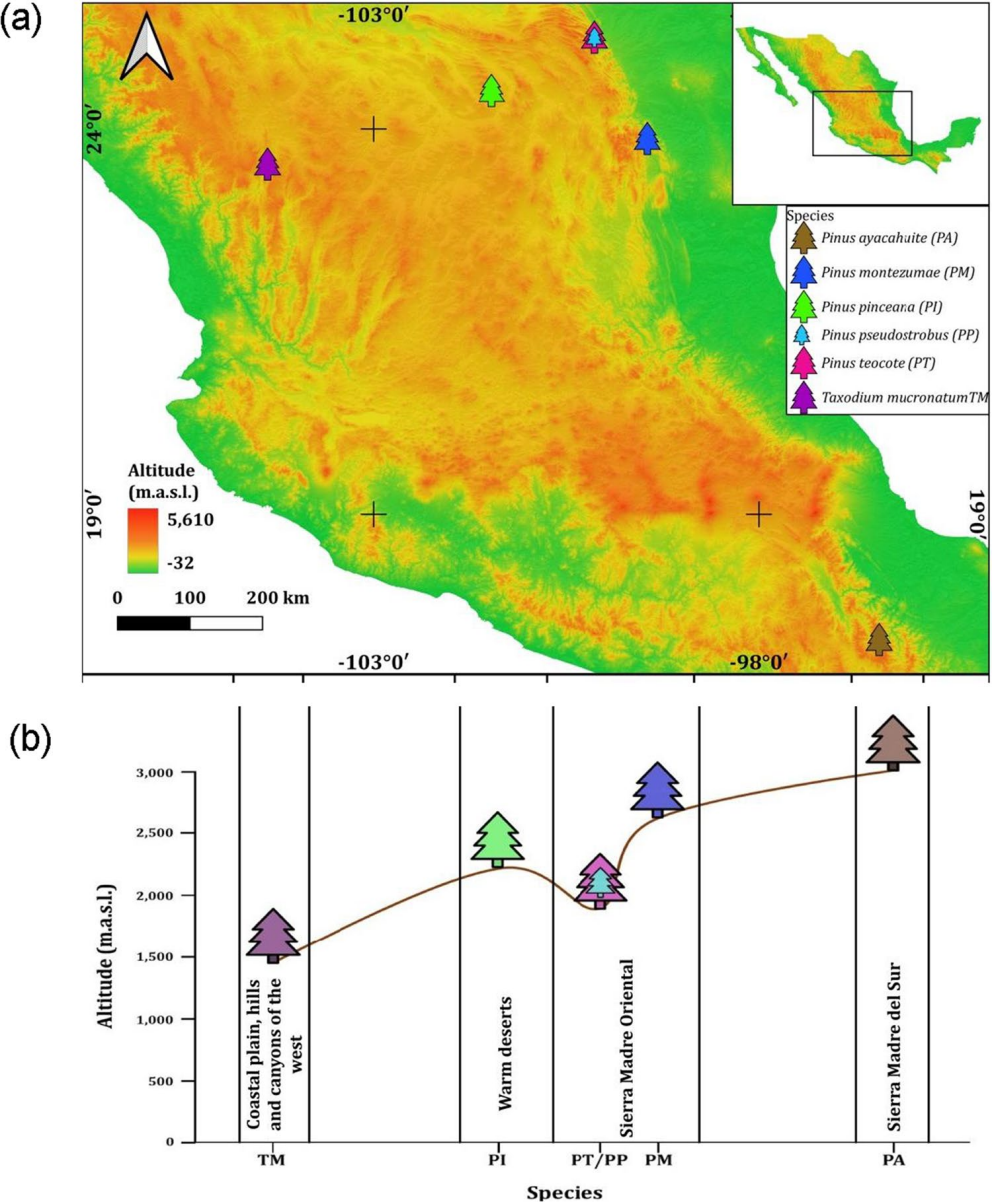
**a** Map showing the location of the young conifer stands studied in Mexico and **b** altitudinal gradient of sampled stands

This study was carried out in six forests located across wide latitudinal (17.38° − 25.19° N) and altitudinal (1434 − 2985 m a.s.l.) gradients in Mexico (Table 1, Fig. 1). Five sampled species were pines (Pinaceae family), and the other species was *Taxodium mucronatum* C. Lawson (Cupressaceae family). All sites correspond to un-even aged, young stands under regular practices of forest management where the selected species was dominant and comprised at least 60% of the stand basal area. Overall, the climate in the study areas was dominated by the summer monsoon leading to dry winters and wet summers (Supporting Information, Fig. S1).

**Table 1:** Geographical, topographical, and climatic features of study sites

Tree species (code)	Latitude (N)	Longitude (W)	Altitude (m a.s.l.)	Mean annual temperature (°C)	Total annual precipitation (mm)
*Pinus teocote* (PT)	25.191	100.131	1900	13.8	744
*Pinus pseudostrobus* (PP)	25.191	100.131	1900	13.8	744
*Pinus pinceana* (PI)	24.499	101.464	2195	15.5	421
*Pinus montezumae* (PM)	23.877	99.446	2579	17.9	730
*Taxodium mucronatum* (TM)	23.545	104.374	1434	17.6	503
*Pinus ayacahuite* (PA)	17.381	96.444	2985	17.5	1002

*Pinus teocote* Schiede ex Schltdl. & Cham. and *Pinus pseudostrobus* Lindl. trees were sampled in the Ejido-La Trinidad site (Nuevo Léon state). Climate in the study area is temperate sub-humid, with an annual precipitation of 700 to 800 mm and average temperature ranging between 12.0 and 16.0 °C. Substrates are lithosol soils and predominant vegetation types are pine and mixed oak-pine forests (INEGI 1986; García-Aranda et al. 2012) . Forests are diverse and contain species of the genera *Cornus*, *Ilex*, *Abies*, *Carya*, *Taxus*, and *Picea* (CONABIO-CONANP 2009). Isolated bark beetle outbreaks have been reported in this site affecting some pine stands since 1991, and droughts and fires occurred in 1998 and 1999 (Hernández-Rodríguez 2019).

*Pinus pinceana* Gordon & Glend. was sampled in a stand located in Mazapil (Zacatecas state), where arid and semiarid climate conditions predominate (SNIARN 2005). The average annual temperature ranges from 15 to 22 °C and annual precipitation ranges from 125 to 450 mm. Vegetation in this area shows diverse adaptations to aridity (Rzedowski 1978), including xerophytic shrubland, halophytic vegetation, and gypsophilous grassland, as well as fragmented pine forests (INEGI 2005). The main soil types are Calcisol and Chernozem, among others (INEGI 2007). Numerous logging activities affected this area as a result of the establishment of mining companies in the region (Panico and Garibay-Orozco 2011).

*Pinus montezumae* Lamb. is situated in Rancho Nuevo (Tamaulipas state). The site has a dominant warm semi-cold climate (INEGI 1986, 2010). The temperature ranges between 12.6 and 18.0 °C with annual precipitation of 700–800 mm (CONAGUA 2010). The area is dominated by lithosol soils, and the predominant vegetation types are pine and mixed oak-pine forests (García-Aranda et al. 2012). A major fire affected this area in 1998 (Hernández-Rodríguez 2019).

The *T. mucronatum* stand is located on a riparian zone near El Mezquital site (Durango state). The climate of this area is semi-dry temperate with an average temperature ranging between 12.0 and 18.0 °C and annual precipitation ranging between 400 and 600 mm. The dominant vegetation is grassland with riparian tree species. The area has feozems, regosol, and cambisol soils (González-Elizondo et al. 2007). Due to the presence of runoff, this area is highly used for agriculture, so trees are subjected to anthropogenic disturbances.

The sampled *Pinus ayacahuite* Ehrenb. ex Schltdl. stand was situated near Ixtlán de Juárez (Oaxaca state). This area is characterized by its heterogeneous geographic relief with slopes and gradients ranging from 40 to 60% and altitudes ranging from 1500 to 3200 m a.s.l. The climate of this area is temperate sub-humid with average annual temperature of 16–20 °C and annual precipitation ranging between 900 and 1700 mm. The soil type is luvisol with a medium loamy texture and clay accumulation in the subsoil. The predominant vegetation consists of pine-oak forests and secondary vegetation of the pine-oak forest (Vásquez-Cortez et al. 2018). This area is under forest harvesting, and the main disturbance is logging (Ramírez-Santiago et al. 2019).

### Climate data

Climate data (mean monthly temperature and total precipitation) were obtained from the TerraClimate dataset gridded at 4-km resolution (Abatzoglou et al. 2018). In addition, two variables related to drought stress were calculated: (1) the vapor pressure deficit (VPD), which reflects the water evaporative demand (Grossiord et al. 2020); and (2) the climate water balance, which reflects changes in soil moisture (Stephenson 1990). The VPD is the difference between water vapor pressure and saturation pressure, and it was calculated from air temperature and relative humidity data following Williams et al. (2013). The water balance is the difference between precipitation and potential evapotranspiration (P-PET), and it was calculated following a modified Thornthwaite method developed by Willmott et al. (1985) and using the AET calculator (available at the webpage https://pages.uoregon.edu/dgavin/software.html).

### Sampling and processing of earlywood and latewood width data

Using a 5-mm Pressler increment borer, we sampled two cores at 1.3 height oriented perpendicular to the slope from 19 to 20 young trees randomly selected near the stand center at each site. To estimate tree age at 1.3 m, we counted the number of annual rings in those cores reaching the pith.

Tree-ring cores were air dried and mounted on wooden frames. The samples were polished using progressively coarse to fine sandpaper, and we synchronized visually the characteristic sequences of radial growth. The earlywood (EW) and latewood (LW) widths were measured using the VELMEX system with 0.001 mm accuracy. Once the measurements were completed, dating was checked using the program COFECHA (Holmes 1983). We constructed EW and LW chronologies (mean series of indices) for each variable and species using the dplR library (Bunn 2008) of the software R (R Core Team 2021) . To detrend the raw EW and LW series, first we fitted negative exponential models which allowed removing age- and disturbance-related influences (Fritts 1976). Then, EW and LW standardized indices were obtained by dividing observed by fitted values. Finally, we removed most of the first-order autocorrelation by fitting autoregressive models and obtained mean series or chronologies by using a bi-weight robust mean. Several statistics were calculated to characterize the EW and LW series including their means and standard deviations. We also calculated the first-order autocorrelation (AC) of raw ring-width data to show the year-to-year persistence in growth and the mean sensitivity (MSx) and mean correlation of ring-width indices between trees (Rbt) to show the year-to-year relative variability in growth and the coherence in growth among trees of the same site and species, respectively (Fritts 1976).

**Fig. 2:**
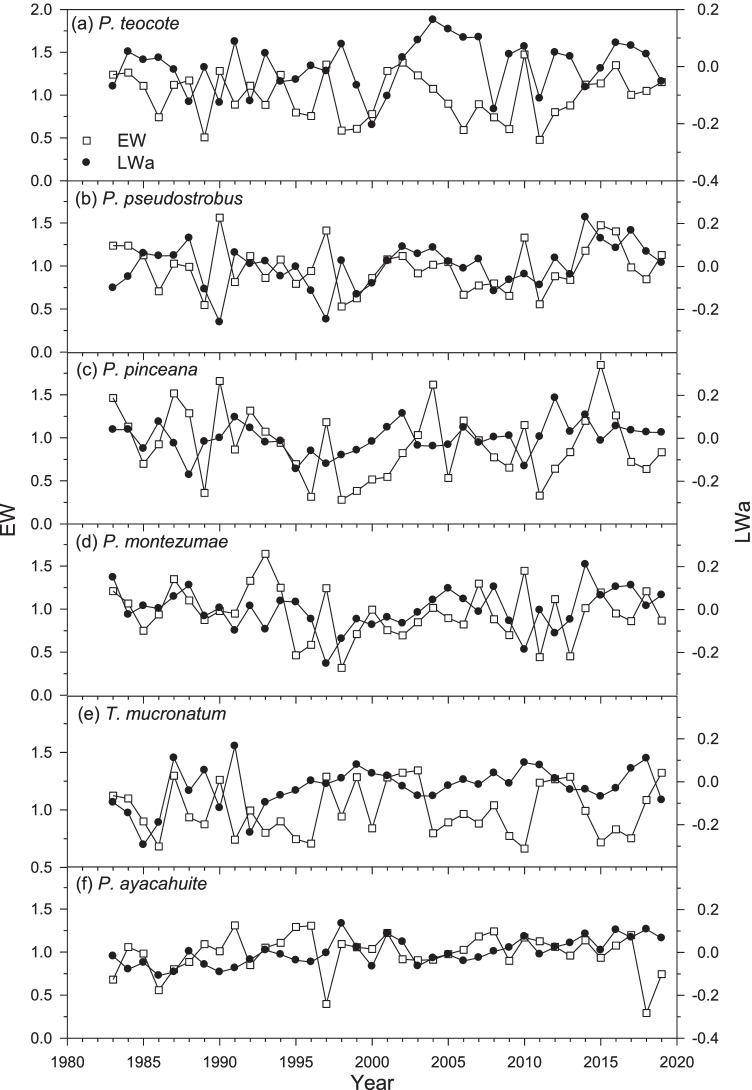
Chronologies of earlywood width (EW, empty squares, left y axes) and adjusted latewood width (LWa, filled circles, right y axes) indices obtained for the six study species and considering the common period 1983–2019

In all study species excepting *T. mucronatum* and *P. teocote*, EW and LW chronologies were positively and significantly (*p* < 0.05) correlated (Fig. 2, Supporting Information, Table S1). Therefore, to remove the dependence of LW on EW we fitted linear regressions and obtained the LW residuals which were named LW adjusted (hereafter LWa) following Meko and Baisan (2001).

Finally, to summarize the relationships among EW and LWa chronologies of all species, a principal component analysis (PCA) was calculated on the variance–covariance matrix of chronologies considering the common period 1983 − 2019. In addition, we also calculated Pearson correlations among the EW and LWa residual chronologies.

### Climate-growth correlations at seasonal scale

We calculated Pearson correlations of EW and LWa residual chronologies with the four monthly climate variables (mean temperature, precipitation, VPD, P-PET) from the previous October up to the current September. This temporal window was chosen based on previous studies on these and similar conifer species growing in Mexico (e.g., Santillán-Hernández et al. 2010; González-Cásares et al. 2017). Correlations were calculated for the common period 1983 − 2019, and the 0.05 and 0.01 significance levels were plotted.

#### VS-Lite model

We used the Vaganov-Shashkin Lite (VS-Lite) model to infer the most limiting climate conditions to radial growth. The VS-Lite model simulates standardized series of tree ring width indices as a function of monthly mean temperature, precipitation, and latitude (Tolwinski-Ward et al. 2011). These inputs are used to estimate growth rates arising from temperature (G_T_) and soil moisture limitations (G_M_). These climate constraints are assumed to limit growth at each monthly time step by mimicking the nonlinear growth responses to climate (Vaganov et al. 2006). The VS-Lite model has been widely tested for parameter estimation and global applicability in dendroclimatology (Tolwinski-Ward et al. 2011, 2013, 2015; Breitenmoser et al. 2014; Jörg et al. 2021) and dendroecology (e.g., Sánchez-Salguero et al. 2017a, b; Sánchez-Salguero and Camarero 2020; Tumajer et al. 2021a, b).

The VS-Lite model was fitted to the EW indexed series of the six study species considering the common period 1983–2019. We obtained the four basic parameters of the model simulations (T_1_, T_2_, M_1_, and M_2_; see TolwinskiWard et al. 2011, 2013). These parameters are bootstrap split sampled and validated using contemporaneous observations and VS-Lite simulations within the considered period. The first temperature (T_1_) and moisture (M_1_) parameters correspond to the thresholds below which growth will not occur, whereas the second parameters (T_2_, M_2_) correspond to the optimal values above which growth is not limited by climate. The growth period was defined as a 16-month interval, from prior September to current December (Tolwinski-Ward et al. 2011). Other parameters such as runoff or root depth were taken from other studies (Evans et al. 2006; Tolwinski-Ward et al. 2011, 2013; Pompa-García et al. 2017). We assumed uniform priors for the growth function parameters and independent, normally distributed errors for EW values evaluated by 10,000 iterations and a white Gaussian noise model error (cf. Tolwinski-Ward et al. 2013).

Finally, we selected calibration (1983 − 2000) and verification (2001 − 2009) periods to test the validity of VS-Lite models. Models were run over the verification period using the parameters calibrated over the calibration period. To test the temporal stability of the models, Pearson correlation coefficients and their significance levels were calculated for each species between observed and simulated EW width indices on both the calibration and the verification periods.

## Results

### Ring-width, EW, and LW statistics

The tree age at 1.3 m ranged between 37 (*T. mucronatum*) and 63 (*P. montezumae*) years (Table 1). Tree-ring chronologies of *Pinus pinceana* showed the lowest first-order autocorrelation, but the highest mean sensitivity and correlation between trees. This suggests a high responsiveness to climate and a high synchrony among co-occurring conspecifics (Table 2). This species also showed the lowest growth rate, whereas *P. teocote* showed the widest rings. A similar ranking was observed in the case of EW. Analogously, *P. pinceana* and *P. ayacahuite* formed the narrowest LW (0.11–0.12 mm), while *P. teocote* and *P. pseudostrobus* formed the widest LW (0.47 mm). On average, the EW comprised 88% of the ring width, with the lowest and highest percentages observed in *T. mucronatum* (82%) and *P. ayacahuite* (96%), respectively. The period common to all species was 1983–2019 (Fig. 2).

**Table 2:** Statistics of the width chronologies calculated on earlywood (EW) and latewood (LW) mean series. Data are means ± standard deviation (SD). Variables’ abbreviations: *AC1*, first-order autocorrelation; *MSx*, mean sensitivity, *Rbt*, mean correlation of ring-width indices between trees

Species (code)	No. trees (no. cores)	Best replicated timespan (no. trees/no. cores)	Ring parameter	Mean ± SD (mm)	AC1	MSx	Rbt
*Pinus teocote* (PT)	20 (40)	1977–2019 (20 / 40)	EW	3.82 ± 2.24	0.59	0.37	0.51
			LW	0.47 ± 0.07			
*Pinus pseudostrobus* (PP)	20 (40)	1983–2019 (20/40)	EW	3.60 ± 1.53	0.63	0.36	0.53
			LW	0.47 ± 0.08			
*Pinus pinceana* (PI)	20 (40)	1959–2019 (20/40)	EW	0.89 ± 0.42	0.35	0.55	0.62
			LW	0.11 ± 0.01			
*Pinus montezumae* (PM)	22 (43)	1957–2019 (20/40)	EW	1.76 ± 1.33	0.63	0.42	0.48
			LW	0.29 ± 0.12			
*Taxodium mucronatum* (TM)	22 (43)	1983–2019 (20/40)	EW	1.11 ± 0.51	0.62	0.30	0.32
			LW	0.24 ± 0.05			
*Pinus ayacahuite* (PA)	20 (40)	1962–2019 (20/40)	EW	2.73 ± 0.71	0.59	0.29	0.33
			LW	0.12 ± 0.01			

### Relationships among EW and LWa chronologies

**Fig. 3:**
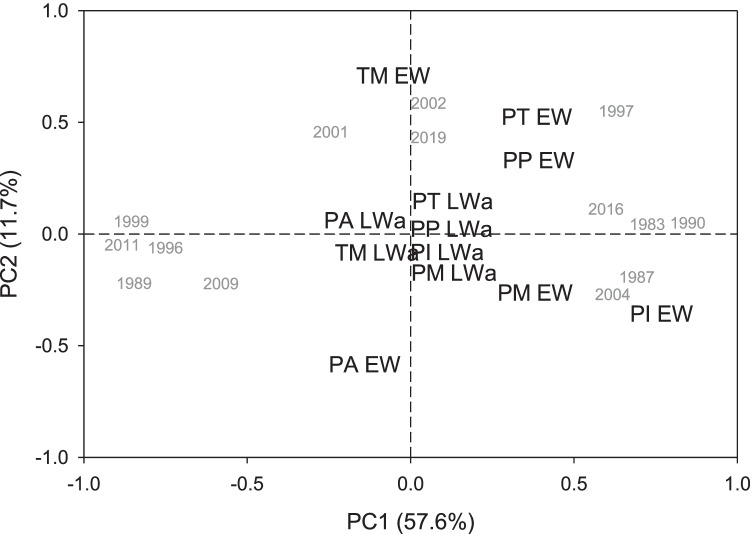
Biplot showing the first (PC1) and second (PC2) principal components of a PCA calculated on the covariance matrix of earlywood (EW) and adjusted latewood (LWa) chronologies (period 1983‒2019). Values between parentheses show the amount of variance explained by PC1 and PC2. Years with extreme PC1 or PC2 scores are presented. Species’ abbreviations: PA, *Pinus ayacahuite*; PI, *Pinus pinceana*; PM, *Pinus montezumae*; PP, *Pinus pseudostrobus*; PT, *Pinus teocote*; and TM, *Taxodium mucronatum*

The first (PC1) and second (PC2) scores of the PCA accounted for 57.6% and 11.7% of the EW and LWa variability, respectively (Fig. 3). The main variables along the PC1 axis were the *P. pinceana* and *P. montezumae* and *P. pseudostrobus* EW chronologies, whereas the main variables along the PC2 axis were the *T. mucronatum* and *P. teocote* EW chronologies. The *P. pinceana* and *P. ayacahuite* EW chronologies presented the most negative PC2 loadings. The years’ scores on the PCA biplot and along the PC1 axis showed years of low growth such as 2011, 1999, 1989, and 1996 and years of high growth such as 1990, 1983, 1987, 1997, 2016, and 2004. On the PC2 axis, we found high scores for years 2001, 2002 and 2019 corresponding to high EW production in *T. mucronatum* and *P. teocote* (Fig. 2).

### Climate-growth relationships

**Fig. 4:**
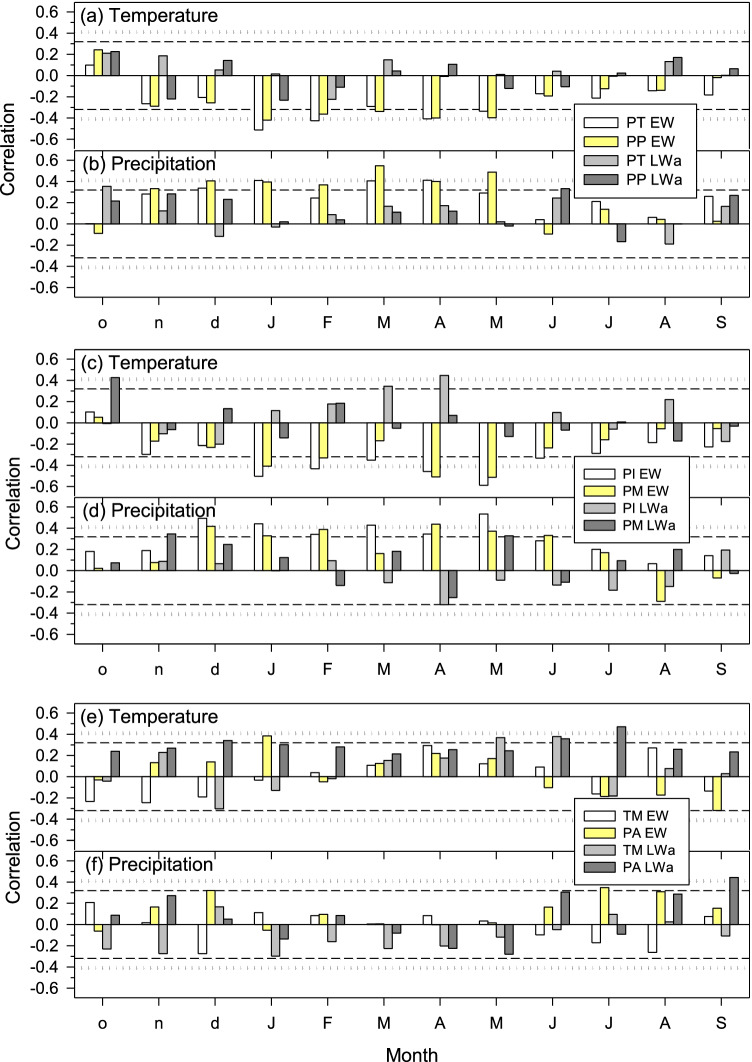
Climate-growth correlations based on measured climate variables (temperature and precipitation) and ring growth chronologies (EW, earlywood; LWa, adjusted latewood). Dashed and dotted horizontal lines show the 0.05 and 0.01 significance levels, respectively. Pearson correlations were calculated for the common period 1983–2019 considering a temporal window from previous October (o) to current September (S). Species’ abbreviations: PA, *Pinus ayacahuite*; PI, *Pinus pinceana*; PM, *Pinus montezumae*; PP, *Pinus pseudostrobus*; PT, *Pinus teocote*; and TM, *Taxodium mucronatum*

Warm conditions negatively affected EW formation of *P. teocote*, *P. pseudostrobus*, *P. pinceana*, and *P. montezumae*, while growing-season precipitation enhanced growth (Fig. 4). However, the EW series of *T. mucronatum* and *P. ayacahuite*, two species which mainly develop in humid sites, showed non-significant (*p* > 0.05) correlations with precipitation (Fig. 4). In these two species, warmer conditions from May to July lead to higher LWa values, while a similar positive association was observed for April temperatures in the case of *P. pinceana*.

**Fig. 5:**
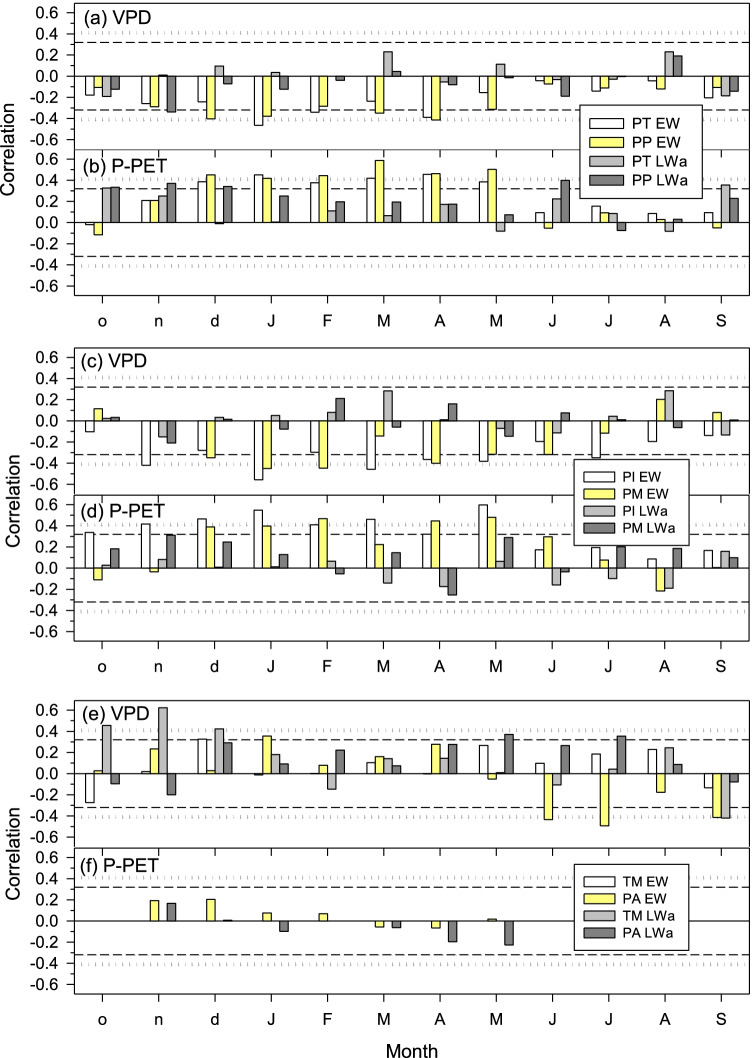
Climate-growth correlations based on calculated climate variables (VPD and water balance, i.e., P-PET) and ring growth chronologies (EW, earlywood; LWa, adjusted latewood). Dashed and dotted horizontal lines show the 0.05 and 0.01 significance levels, respectively. Pearson correlations were calculated for the common period 1983–2019 considering a temporal window from previous October (o) to current September (S). Species’ abbreviations: PA, *Pinus ayacahuite*; PI, *Pinus pinceana*; PM, *Pinus montezumae*; PP, *Pinus pseudostrobus*; PT, *Pinus teocote*; and TM, *Taxodium mucronatum*

All species’ EW indices, excepting those of TM, were negatively affected by VPD (Fig. 5). High VPD from December to May was associated to a lower EW production for *P. teocote*, *P. pseudostrobus*, and *P. pinceana*, but in the case of *P. ayacahuite* such limitation occurred in June and July.

### Climatic constraints of earlywood production inferred from the VS-Lite simulations

**Fig. 6:**
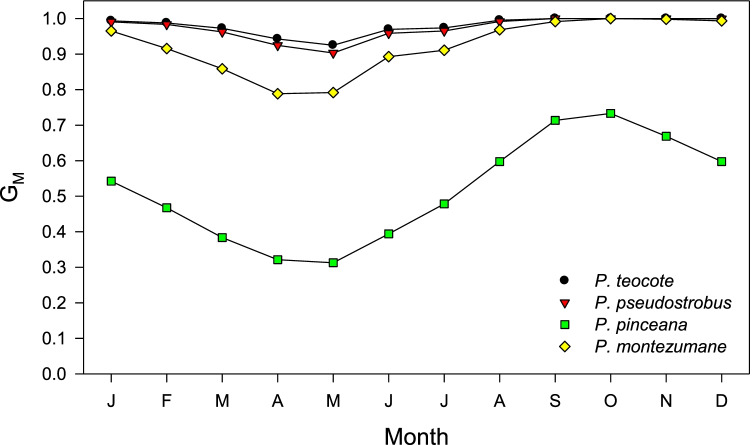
VS-Lite modeling results showing monthly values of the G_M_ function which describes tree growth limitations by soil moisture. Only pine species with significantly fitted models are plotted (see Table 3)

The VS-Lite model was able to simulate the variability of EW indices in four out of the six study species (Table 3). In the case of *T. mucronatum* and *P. ayacahuite*, the two species showing the lowest EW responsiveness to climate variables (Figs. 5 and 6), the observed and simulated EW width indices presented positive but non-significant (*p* > 0.05) correlations (Table 3). The highest correlation between observed and simulated EW indices was found for *P. pinceana*, the species showing the highest limitation of EW formation by low soil moisture according to the G_M_ function. Such limitation peaked in May in this species and also in *P. teocote* and *P. pseudostrobus*, buy it did in April in the case of *P. montezumane* (Table 3, Fig. 6). The VS-Lite models of EW indices were stable through time with significant Pearson correlations in the calibration and verification periods (Table S2).

**Table 3:** VS-Lite statistics of the models fitted to mean series of earlywood width (EW) indices. The growth response parameters (T_1_, T_2_, M_1_, and M_2_ for minimum and optimal temperature and soil moisture values, respectively) are shown. The relative volumetric soil moisture content is the relation between the volume of water and the soil volume (v/v)

Tree species (code)	Correlation observed vs. simulated EW indices	*p*	T_1_ (ºC)	T_2_ (ºC)	M_1_ (v/v)	M_2_ (v/v)
*P. teocote* (PT)	0.67	< 0.001	7.48	11.11	0.024	0.145
*P. pseudostrobus* (PP)	0.66	< 0.001	5.54	11.40	0.004	0.142
*P. pinceana* (PI)	0.73	< 0.001	6.91	11.82	0.004	0.237
*P. montezumae* (PM)	0.54	0.0006	3.54	12.85	0.031	0.167
*T. mucronatum* (TM)	0.15	0.4651	6.23	12.73	0.011	0.100
*P. ayacahuite* (PA)	0.25	0.1374	6.59	13.74	0.059	0.499

**Fig. 7:**
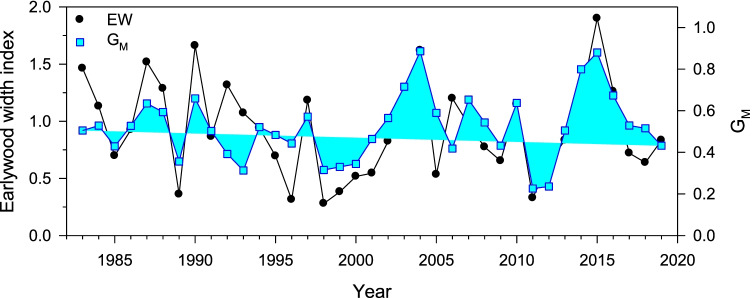
Yearly values of the earlywood (EW) chronology measured in *P. pinceana* and the G_M_ function, which quantifies growth limitations by low soil moisture. Both series were positively correlated (*r* = 0.71, *p* < 0.001)

In the case of *P. pinceana*, the species with best VS-Lite fit (Table 3), the EW chronology, and the G_M_ series, depicting growth limitation by soil moisture, were highly correlated (*r* = 0.71, *p* < 0.001; Fig. 7).

## Discussion

We found that only four species showed growth limitations attributed to soil moisture, particularly the species inhabiting the driest sites (*P. pinceana*), while two of them from mesic sites (*P. ayacahuite* and *T. mucronatum*) were not responsive to changes in soil moisture, and we were unable to model how climate constrained their earlywood production. It seems that soil moisture constraints and maximum temperatures from winter to spring are key elements that modulate the plasticity of intra-annual radial growth in the study Mexican conifers. The multi-species perspective helped to capture climatic constraints experienced by each species in each particular site (González-Cásares et al. 2017).

The seasonal resolution reduces uncertainty in quantifying the responses of young tree stands to climate when they are subjected to different aridity degree. Considering earlywood and latewood production provided new and suitable proxies for estimating climate vulnerability by disentangling different processes such as hydraulic conductivity, mostly accounted for by earlywood, and carbon fixation in cell walls, mostly accounted for by latewood (Domec and Gartner 2002; Domec et al. 2009; Björklund et al. 2017; Szejner et al. 2018). As expected, earlywood is directly related to drought stress (decreased relative humidity, increased water demand, lower soil moisture) since it is a portion of the ring very sensitive to xylem embolism, and it is formed when the rate of production of radially enlarging cells peaks (Vaganov et al. 2006; Domec et al. 2009; Pasho et al. 2012; Cuny et al. 2018).

Earlywood production was limited by spring drought, particularly by elevated evaporative water demand. The VPD is non-linearly and positively related to temperature, so rapidly warmer conditions could amplify water loss through leaves and cascade on growth decline and forest dieback as observed in semi-arid regions from the southwestern USA (Williams et al. 2013). Warmer conditions could also lengthen the growing season and increase growth rates whenever water availability is not limiting (Cornes et al. 2017).

Overall, we observed two different patterns in terms of climatic constraints of growth: (1) *P. teocote*, *P. pseudostrobus*, and *P. montezumane* show less limiting climatic factors in the high-altitude, mesic sites they usually inhabit, while (2) *P. pinceana*, abundant in xeric sites characterized by low productivity and high inter-annual precipitation variability (Villarreal-Quintanilla et al. 2009), is a species sensitive to dry conditions. The importance of the previous winter climate as a driver of growth in the next growing season has been well documented for these and other drought-prone forest ecosystems (Vivar-Vivar et al. 2021). The role played by winter rainfall could be explained because of (i) the refilling of soil pools or because of (ii) an enhanced production of carbohydrates as inferred for Mediterranean pine forests subjected to dry summer conditions (Camarero et al. 2010). These two effects explain carryover effects from prior growth to current earlywood and from current earlywood to current latewood which justifies the analysis of LWa chronologies (e.g., Acosta-Hernández et al. 2018).

The positive association found between summer temperature and LWa in the two species from mesic sites (*T. mucronatum* and *P. ayacahuite*) can be explained by the triggering of latewood formation due to warmer summer conditions inhibiting cell expansion (Lupi et al. 2010). Thus, temperature is a main driver of forest productivity in mesic sites, which agrees with the decay of drought impact on tree growth as we move from xeric to mesic regions (Pompa-García et al. 2021). In short, *T. mucronatum* and *P. ayacahuite* take advantage of warm and humid site conditions to grow and form stem wood. Excepting *T. mucronatum*, the growth of the other analyzed species was negatively impacted by water shortage, which allows forecasting growth decline or canopy dieback in response to severe droughts in some marginal populations of those species, i.e., those forming the climatic distribution limits (Sánchez-Salguero et al. 2017a).

Although our analyses did not include data on cambial dynamics (xylogenesis), which we will consider in further research, the presented findings suggest that an adequate level of soil moisture enhances radial growth by rising maximum rates of cell enlargement and division in spring rather than by lengthening the growing season (Tumajer et al. 2021a). Seasonal changes in water availability (soil moisture) control ring growth modifying the proportion between earlywood and latewood in the rings (Domec and Gartner 2002). Therefore, and as we hypothesized, hydraulic conductivity is proportional to earlywood production, given their primary role as driver of hydraulic conductivity. This agrees with the observed decreased of lumen area in earlywood tracheids in response to increasing aridity (Cuny et al. 2018). This is also in line with the negative association found between earlywood production and VPD showing how evaporative demand leads to dry soil conditions constraining growth (Fig. 5). Summer monsoon rains could also alleviate part of spring drought stress, particularly during the late growing season (Pompa-García and Antonio-Némiga 2015), but temporal lags between water shortage and xylogenesis make challenging to test this hypothesis (Belmecheri et al. 2018). In the dry regions of western North America, inter- and intra-annul precipitation variability is high leading to notable changes in soil moisture and affecting xylogenesis through growth plasticity including changes in earlywood production and the formation of intra-annual density fluctuations (Ziaco et al. 2018). Additional studies should disentangle how xylogenesis contributes to the different responses of seasonal ring growth to soil water availability in young trees of coexisting species and across environmental gradients. Overall, growth models allow disentangling the mechanisms leading to the transition from earlywood to latewood (Cartenì et al. 2018) and better understanding intra-annual growth patterns and xylogenesis (Buttò et al. 2020).

The arid environment that faces *P. pinceana* corresponds to low soil moisture and elevated evaporative water demand (high VPD), two drought components which decrease cell turgor and cambial division constraining the production of more and wider earlywood cells (Carvalho et al. 2015; Rathgeber et al. 2016). These processes lead to lower radial growth and hydraulic conductivity explaining the elevated sensitivity of *P. pinceana* earlwyood production to low spring soil moisture.

Contrasting patterns among species regarding climatic constraints were observed, with strong earlywood limitations by low soil moisture in spring. Consequently, simulations showed conditions that climatically dominate the altitudinal gradients: from the arid, drought-stressed *P. pinceana* (which presented the strongest simulations of EW) to mesic, high-elevation sites where low soil moisture was not constraining growth such as in the case of *P. pseudostrobus* and *P. teocote*, while *P. montezumae* occupied an intermediate position.

Our results suggest the need to study additional aspects not considered by the VS-Lite model, which is based on non-linear relationship between climatic limiting factors and cambial activity (Vaganov et al. 2006). For example, it is necessary to complement these simulations with studies of even better temporal resolution of wood development (e.g., xylogenesis data at weekly to biweekly scales; e.g., Buttò et al. 2020) and forest productivity (e.g., remote sensing data). Xylogenesis series assessing intra-annual growth dynamics for several years should allow quantifying the relative roles played by growth rates or growing seasons in tree growth, and they could be used to constrain growth simulations (e.g., Tumajer et al. 2021b).

## Conclusions

Our dendroclimatic study conducted on young trees pointed out new insights of drought stress on seasonal radial growth. The intra-annual analysis of radial growth of six Mexican conifers revealed how temperature and precipitation are affecting seasonal ring formation of these species. Wet and cool conditions in the previous winter and current spring enhance earlywood production, particularly in sensitive species inhabiting dry sites such as *P. teocote*, *P. pseudostrobus*, *P. pinceana*, and *P. montezumae*. Thus, measuring separately earlywood and latewood improves our understanding of growth-drought relationships. In particular, earlywood width is a strong proxy of spring soil moisture in species from xeric regions (e.g., *P. pinceana*). Growth is limited by low soil moisture and an elevated evaporative water demand from winter to spring. Latewood formation is enhanced by warmer summer conditions in the two species inhabiting mesic sites (*P. ayacahuite*, *T. mucronatum*), which suggests uncoupling between the climatic factors limiting earlywood and latewood production. It could be forecasted that some tree species would be modifying their seasonal growth responses to drought under more arid conditions.

## Supplementary Information

Below is the link to the electronic supplementary material.

Supplementary file1 (DOCX 129 KB)
