# Supplementary material for: Climate sensitivity of seasonal radial growth in young stands of Mexican conifers
Source: Int J Biometeorol. 2022 Jun 8;66(8):1711–23. doi: 10.1007/s00484-022-02312-3 (PMC9300551; doi:10.1007/s00484-022-02312-3)
Supplement: Supplementary file 1 — Supplementary file1 (DOCX 129 KB) [file 484_2022_2312_MOESM1_ESM.docx]

**Supporting Information**

**Table S1.** Pearson correlations (common period 1983‒2019) calculated among residual chronologies of earlywood (EW) and latewood (LW) width indices. Species’ abbreviations: PA, *Pinus ayacahuite*; PI, *Pinus pinceana*; PM, *Pinus montezumae*; PP, *Pinus pseudostrobus*; PT, *Pinus teocote*; and TM, *Taxodium mucronatum*.

|  | PM EW | PM LW | PI EW | PI LW | PP EW | PP LW | PT EW | PT LW | PA EW | PA LW | TM EW | TM LW |
| --- | --- | --- | --- | --- | --- | --- | --- | --- | --- | --- | --- | --- |
| PM EW |  | 0.00 | 0.00 | 0.01 | 0.01 | 0.11 | 0.00 | 0.03 | 0.07 | 0.22 | 0.33 | 0.99 |
| PM LW | 0.62 |  | 0.00 | 0.00 | 0.02 | 0.00 | 0.03 | 0.33 | 0.34 | 0.49 | 0.14 | 0.24 |
| PI EW | 0.58 | 0.60 |  | 0.00 | 0.00 | 0.01 | 0.00 | 0.01 | 0.10 | 0.11 | 0.23 | 0.01 |
| PI LW | 0.40 | 0.51 | 0.67 |  | 0.00 | 0.00 | 0.02 | 0.04 | 0.26 | 0.45 | 0.59 | 0.07 |
| PP EW | 0.45 | 0.39 | 0.68 | 0.45 |  | 0.01 | 0.00 | 0.00 | 0.27 | 1.00 | 0.52 | 0.07 |
| PP LW | 0.27 | 0.52 | 0.44 | 0.47 | 0.43 |  | 0.00 | 0.00 | 0.88 | 0.07 | 0.32 | 0.06 |
| PT EW | 0.48 | 0.35 | 0.59 | 0.38 | 0.88 | 0.53 |  | 0.00 | 0.13 | 0.63 | 0.73 | 0.14 |
| PT LW | 0.35 | 0.16 | 0.44 | 0.33 | 0.55 | 0.57 | 0.71 |  | 0.25 | 0.46 | 0.65 | 0.09 |
| PA EW | -0.30 | -0.16 | -0.27 | -0.19 | -0.19 | -0.03 | -0.25 | -0.19 |  | 0.17 | 0.06 | 1.00 |
| PA LW | -0.21 | -0.12 | -0.27 | -0.13 | 0.00 | 0.30 | 0.08 | 0.12 | 0.23 |  | 0.96 | 0.20 |
| TM EW | -0.20 | -0.30 | -0.24 | 0.11 | -0.13 | -0.20 | 0.07 | -0.09 | -0.37 | 0.01 |  | 0.82 |
| TM LW | 0.00 | -0.24 | -0.52 | -0.36 | -0.37 | -0.37 | -0.29 | -0.34 | 0.00 | 0.26 | 0.05 |  |

**Table S2.** Statistics describing the calibration and verification periods (1983−2000 and 2001−2009, respectively) applied to the VS-Lite models fitted to earlywood width indices in four Mexican conifers. Statistics include Pearson correlations (*r*) and associated significance levels (*p*). The growth response parameters (T_1_, T_2_, M_1_, and M_2_ for minimum and optimal temperature and soil moisture values, respectively) are also shown.

| Period | Tree species (code) | *r* | *p* | T_1_ (ºC) | T_2_ (ºC) | M_1_ (v/v) | M_2_ (v/v) |
| --- | --- | --- | --- | --- | --- | --- | --- |
| Calibration (1983−2000) | *P. teocote* (PT) | 0.778 | 0.000 | 6.958 | 16.726 | 0.001 | 0.155 |
|  | *P. pseudostrobus* (PP) | 0.765 | 0.000 | 7.246 | 16.619 | 0.011 | 0.146 |
|  | *P. pinceana* (PI) | 0.748 | 0.000 | 6.975 | 18.486 | 0.039 | 0.445 |
|  | *P. montezumane* (PM) | 0.736 | 0.001 | 7.427 | 12.865 | 0.002 | 0.158 |
| Verification (2001−2019) | *P. teocote* (PT) | 0.633 | 0.004 | 6.141 | 11.367 | 0.027 | 0.133 |
|  | *P. pseudostrobus* (PP) | 0.656 | 0.002 | 5.070 | 22.090 | 0.063 | 0.223 |
|  | *P. pinceana* (PI) | 0.739 | 0.000 | 7.070 | 11.083 | 0.022 | 0.252 |
|  | *P. montezumane* (PM) | 0.668 | 0.002 | 3.213 | 11.130 | 0.052 | 0.159 |

| (a) |
| --- |
| 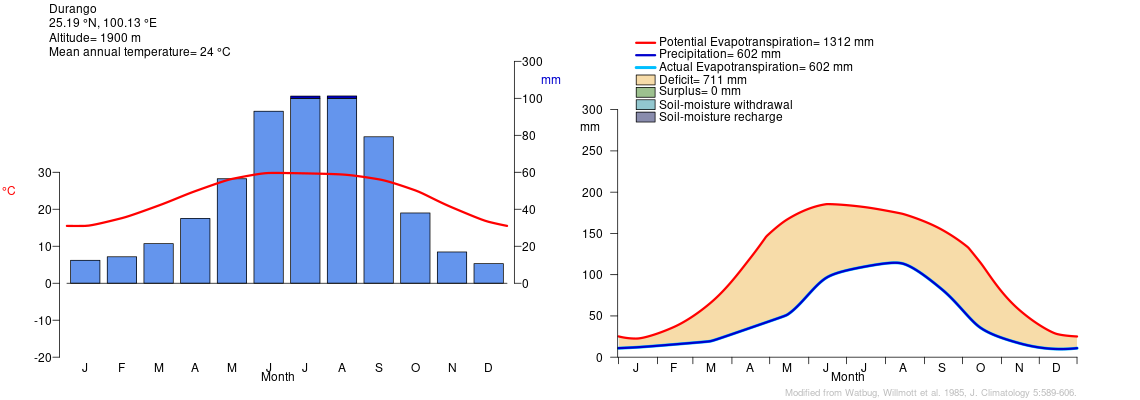 |
| (b) |
| 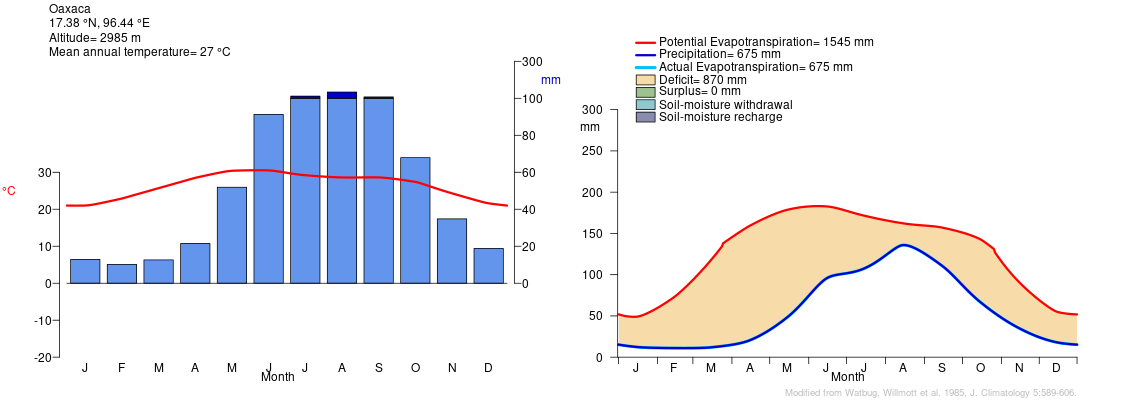 |

**Figure S1.** Climate diagrams and estimated climate water balance in the northernmost (a, *Pinus teocote* and *Pinus pseudostrobus*) and southernmost (b, *Pinus ayacahuite*) sites.
